# Original insights on thrombospondin-1-related antireceptor strategies in cancer

**DOI:** 10.3389/fphar.2015.00252

**Published:** 2015-10-29

**Authors:** Albin Jeanne, Christophe Schneider, Laurent Martiny, Stéphane Dedieu

**Affiliations:** ^1^Laboratoire SiRMa, UFR Sciences Exactes et Naturelles, Université de Reims Champagne-ArdenneReims, France; ^2^CNRS, Matrice Extracellulaire et Dynamique Cellulaire, UMR 7369Reims, France; ^3^SATT NordLille, France

**Keywords:** TSP-1, CD47, CD46, cancer, angiogenesis, innovative therapeutic strategies

## Abstract

Thrombospondin-1 (TSP-1) is a large matricellular glycoprotein known to be overexpressed within tumor stroma in several cancer types. While mainly considered as an endogenous angiogenesis inhibitor, TSP-1 exhibits multifaceted functionalities in a tumor context depending both on TSP-1 concentration as well as differential receptor expression by cancer cells and on tumor-associated stromal cells. Besides, the complex modular structure of TSP-1 along with the wide variety of its soluble ligands and membrane receptors considerably increases the complexity of therapeutically targeting interactions involving TSP-1 ligation of cell-surface receptors. Despite the pleiotropic nature of TSP-1, many different antireceptor strategies have been developed giving promising results in preclinical models. However, transition to clinical trials often led to nuanced outcomes mainly due to frequent severe adverse effects. In this review, we will first expose the intricate and even sometimes opposite effects of TSP-1-related signaling on tumor progression by paying particular attention to modulation of angiogenesis and tumor immunity. Then, we will provide an overview of current developments and prospects by focusing particularly on the cell-surface molecules CD47 and CD36 that function as TSP-1 receptors; including antibody-based approaches, therapeutic gene modulation and the use of peptidomimetics. Finally, we will discuss original approaches specifically targeting TSP-1 domains, as well as innovative combination strategies with a view to producing an overall anticancer response.

## State of the Art

In view of the relatively short-lived benefits observed in targeted therapies that aim at facing advanced primary cancers, the current main therapeutic challenge is to identify original molecular targets in order to limit tumor burden without allowing resistance acquisition (van Beijnum et al., 2015). Indeed, the advent of systems biology over recent years underlined the limits of therapeutic agents designed to block a single pathway and/or growth factor, inevitably leading to the activation of compensatory mechanisms which allow tumor escape and restore disease progression (Wilson et al., 2015). To face the complexity and massive redundancy of signaling pathways and regulatory processes underlying tumor progression, growing attention is accorded to matricellular proteins and their cell receptors as they function as multiple integrators of tumor progression signals at the tumor/microenvironment interface (Murphy-Ullrich and Sage, 2014). According to their definition first given by Paul Bornstein 25 years ago, matricellular proteins regulate a wide range of both malignant and stromal cell functions through interactions with cell-surface receptors or by acting in a coordinated manner with other ECM components or soluble molecules (Sage and Bornstein, 1991). TSPs may be regarded as the archetypes of the matricellular protein group, with TSP-1 first identified from human platelets in 1978 (Lawler et al., 1978).

Thrombospondin-1 is considered a main actor within a tumor microenvironment, while it exerts intricate and sometimes opposite effects on tumor progression. Elevated circulating levels of TSP-1 were early observed in patients presenting breast, lung, gastrointestinal or even gynecological malignancies (Tuszynski et al., 1992; Nathan et al., 1994). In patients receiving myelosuppressive anti-cancer chemotherapy, TSP-1 blood concentrations assessments strongly correspond with platelet counts (Starlinger et al., 2010). Of note, such correlation is also observed in non-malignant processes where platelet activation is high, such as sickle cell disease (Novelli et al., 2012). Nevertheless, others have noted that elevation of circulating TSP-1 in a cancer setting could be even noticed in absence of plasma contamination by platelet activation (Byrne et al., 2007), thus suggesting that TSP-1 plasma levels might originate from sources other than platelets. Therefore, additional work is needed to determine the exact origins of high TSP-1 plasma concentrations, particularly as many non-platelet sources are known to produce TSP-1 such as endothelial cells, cancer cells, or even circulating immune cells (Dawes et al., 1988). Among the range of possibilities, tumor-originating TSP-1 may provide a plausible explanation to elevated levels detected in patients. Indeed, increased TSP-1 mRNA and/or protein levels were observed within the stromal compartment of breast and gastric carcinoma (Clezardin et al., 1993; Brown et al., 1999; Lin et al., 2012). On the contrary, carcinoma cells express undetectable to low levels of TSP-1 in these studies, and loss of TSP-1 expression by cancer cells is described as an important feature of the “angiogenic switch” in a wide range of solid tumors (Naumov et al., 2006). Indeed, TSP-1 expression is typically down-regulated by oncogenes whereas it is promoted by tumor suppressor genes such as p53 (Dameron et al., 1994). Recently, oncogenic Ras was confirmed as being likely to induce phosphorylation of Myc, thus leading to TSP-1 repression and acquisition of an angiogenic phenotype (Watnick et al., 2015). While TSP-1 expression is lost during malignant progression in a wide variety of major cancer types, a few exceptions need nevertheless to be considered. By way of example, TSP-1 is over-expressed by invasive and metastatic melanoma cells, in which it actively contributes to an epithelial-to-mesenchymal transition program (Jayachandran et al., 2014; Borsotti et al., 2015).

Like other matricellular proteins, TSP-1 is a multi-modular and multifunctional protein able to bind a wide variety of ligands, thus considerably increasing the complexity of its translational potential. As a consequence, it seems obvious that strategies blindly targeting a specific function such as gross matricellular protein silencing, or the use of blocking antibodies, may induce severe adverse effects. Indeed the beneficial properties of the multifunctional protein may also be lost under such treatment. Here, we will focus on TSP-1 and two of its receptors viz. CD47 and CD36, to review pre-clinical and clinical outcomes that could be achieved with current developments. Then, we will discuss future directions to target these cell-surface receptors, using small molecules and peptides able to interfere with TSP-1/CD47 as well as TSP-1/CD36 signaling axis in order to reach an overall anticancer response.

## TSP-1: A Main Actor Within Tumor Microenvironment

Thrombospondin-1 has long been considered to play a role in tumor progression; several studies carried out 20 years ago found it to be overexpressed within tumor stroma and in high circulating levels in several cancers (Qian and Tuszynski, 1996; Bertin et al., 1997; Brown et al., 1999). TSP-1 was also reported to contribute to metastatic spread by promoting tumor cell emboli formation (Incardona et al., 1995). In recent years, an increasing number of studies have tended to present TSP-1 as a poor prognosis and recurrence marker in many cancer types including glioma (Perez-Janices et al., 2015), melanoma (Borsotti et al., 2015) as well as ovarian and pancreatic carcinomas (Lyu et al., 2013; Nie et al., 2014; Pinessi et al., 2015). Among TSP-1 ligands, CD36 and CD47 cell-surface receptors act as key integrators of multiple signals regulating tumor growth and dissemination both positively and negatively. Indeed, TSP-1/CD36/CD47 trimolecular signaling platform dynamics as well as interactions involving co-receptors and soluble ligands exert pleiotropic activities on cancer progression, by directly modulating cancer cells behavior or by acting on tumor microenvironment stromal cells (Kazerounian et al., 2008; Sick et al., 2012).

### Modulation of Angiogenesis by TSP-1

Thrombospondin-1 is widely known as an endogenous inhibitor of angiogenesis by negatively regulating NO-mediated signaling in endothelial cells, vSMC and platelets (Isenberg et al., 2008a, 2009b). TSP-1 inhibition of NO/cGMP-related pathways and subsequent antiangiogenic activities are mediated by its interaction with two cell-surface receptors: CD47 and CD36 (Isenberg et al., 2009c; Zhang et al., 2009). CD47 is a ubiquitous 50 kDa receptor consisting of a single N-terminal IgV extracellular domain, five membrane-spanning segments and a short C-terminal cytoplasmic tail (Sick et al., 2012). Although it is now commonly referred to by its immunological marker name, CD47 receptor was first identified through its association with α_V_β_3_ integrin, therefore justifying its former name IAP (integrin associated protein; Brown et al., 1990). At the same time, the ovarian tumor antigen OA3 was characterized (Campbell et al., 1992) and subsequently shown to be the same protein as CD47 (Mawby et al., 1994). CD47 is widely considered as a marker of “self,” and is therefore highly expressed by circulating hematopoietic stem cells, erythrocytes and many malignant cells (Oldenborg et al., 2000; Jaiswal et al., 2009). Notably, CD47 was described as a marker of tumor-initiating cells in leukemia as well as in bladder and liver cancer (Chan et al., 2009; Willingham et al., 2012a; Lee et al., 2014). In cancer, CD47 acts as a “don’t eat me” signal by engaging its macrophage phagocytic counter-receptor SIRPα (signal regulatory protein alpha; Vernon-Wilson et al., 2000; Chao et al., 2012). Thus, CD47 binding to SIRPα present on immune cells causes a dephosphorylation cascade avoiding synaptic myosin accumulation and thereby preventing engulfment (Tsai and Discher, 2008). However, broad evidence now sustains that CD47 signaling functions go well beyond this simple antiphagocytic passive role, with CD47 acting as a sensor for cell–cell and cell–microenvironment signals. Indeed SIRPα can interact with CD47 receptors in *cis* or in *trans*, and CD47/SIRPα signaling should not be considered as unidirectional in so far as SIRPα binding can in turn affect intracellular signaling through CD47 (Latour et al., 2001), which has further been called “reverse” signaling (Sarfati et al., 2008). While this provides an exciting area for future research, numerous studies of CD47 signaling functions that have been published so far focused on CD47 activation by TSP-1. Indeed, TSP-1 available within the ECM is a key regulator of CD47 signaling. CD47 ligation by TSP-1 C-terminal domain dissociates its constitutive association with VEGFR2 and allows inhibition of both early eNOS-activating signals and NO-independent VEGFR2 signaling, thus leading to subsequent antiangiogenic responses (Kaur et al., 2010; Soto-Pantoja et al., 2015). Remarkably, TSP-1:CD47 interaction also redundantly inhibits NO signaling at the level of such downstream effectors as soluble guanylate cyclases (sGC; Isenberg et al., 2006) and cGMP-dependent protein kinases (cGK; Isenberg et al., 2008a,c). Of note, co-immunoprecipitations experiments were recently conducted identifying for the first time TSP-1 as a new ligand for SIRPα, which may result in stimulation of SIRPα phosphorylation and downstream signaling in non-phagocytic cells (Yao et al., 2014). While this process is likely to involve the joint contribution of CD47, it raises the exciting likelihood of a CD47-independent SIRPα signaling under TSP-1 ligation. As cell-free binding assays indicated that TSP-1:SIRPα interaction does not imply the C-terminal domain of TSP-1, further studies considering recombinant fragments as well as molecular docking experiments would be of particular interest to better characterize this newly identified interaction. To further increase the complexity of TSP-1/CD47/SIRPα signaling axis, it has to be noted that both CD47 and SIRPα ectodomains could be target of sheddases and thus provide additional ligands for TSP-1, CD47, and SIRPα (Ohnishi et al., 2004; Maile et al., 2008; Toth et al., 2013).

In addition to CD47-induced effects, a central region of TSP-1 called 3TSR (three TSP-1 type 1 repeats) binds itself to the CD36 membrane receptor, also leading to angiogenesis inhibition. CD36, first identified from platelets as glycoprotein IV (GpIV; Clemetson et al., 1977), is a class B scavenger receptor (Calvo et al., 1995) also acting as fatty acid translocase (Pohl et al., 2005). It is mostly expressed by microvascular endothelial cells and vSMC (Dawson et al., 1997; Silverstein and Febbraio, 2009) in which TSP-1 ligation promotes CD36 association with β1 integrin and VEGFR2 dimer in a tripartite complex, resulting in decreased VEGFR2 phosphorylation under VEGF stimulation (Zhang et al., 2009). Besides, TSP-1 binding to CD36 inhibits NO-related signaling at the level of eNOS by preventing myristate uptake and also its downstream effects. Indeed, both TSP-1 and a peptide derived from the 3TSR as well as a CD36 “agonist” mAb are able to modulate the fatty acid translocase activity of CD36 by preventing myristate uptake in vascular cells (Isenberg et al., 2007). As CD36 is expected to be the main cell membrane protein involved in fatty acid uptake (Koonen et al., 2005), one should bear in mind that targeting this receptor may affect lipoprotein and glucose metabolism, and therefore lead to cardiovascular complications. In humans, CD36 deficiency is associated with phenotypic expression of the “metabolic syndrome,” i.e., hypercholesterolemia, hyperglycemia, insulin resistance, and higher blood pressure (Hirano et al., 2003). Besides, TSP-1 and TSP-1-derived agents that inhibit myristate uptake through CD36 activation are also likely to affect non angiogenesis-related signaling pathways as post-translational myristoylation regulates many protein and cell functions (Martin et al., 2011). While TSP-1 is a high affinity ligand for CD47, binding to CD36 requires higher concentrations that overcome physiological levels. Apart from this 100-fold difference in binding affinities, results from null cells and animals also indicate that while TSP-1 ligation of either CD36 or CD47 is sufficient to inhibit NO-stimulated vascular responses, only CD47 is necessary for such TSP-1 activity at physiological concentrations (Isenberg et al., 2006). However, considering that TSP-1 protein levels in tumor and surrounding tissue are found to be elevated in several cancers (for review, see Kazerounian et al., 2008), one can assume that CD36-activating concentrations are reached within a tumor microenvironment.

### TSP-1 Direct Impact on Cancer Cell Behavior and Tumor Immunity

Far from being restricted to angiogenesis modulation, the effects of TSP-1 on tumor progression are multifaceted and sometimes even opposite depending on the molecular and cellular composition of the microenvironment. Indeed, its ability to interact with multiple ligands enables TSP-1 to regulate a wide range of processes such as tumor cell adhesion (Li et al., 2006), proliferation (Sick et al., 2011), survival or apoptosis (Manna and Frazier, 2004; Saumet et al., 2005; Rath et al., 2006a,b), tumor invasion and metastatic dissemination (Jayachandran et al., 2014; Borsotti et al., 2015), inflammation, immune response (Grimbert et al., 2006) and response to treatment (Lih et al., 2006; Bi et al., 2014). Such pleiotropic effects may be governed by TSP-1 concentration as well as by its origin, whether it originates from tumor cells or the stroma compartment (Pinessi et al., 2015). Reverse responses may also be observed depending on the cancer type. For instance, CD47 ligation by TSP-1 induces killing of breast cancer cells (Manna and Frazier, 2004) while it was reported to inhibit apoptosis and promote drug resistance in thyroid carcinoma cells (Rath et al., 2006a,b). Besides, TSP-1 can also trigger cancer cell death by interacting with the CD36 receptor as recombinant 3TSR fragments of TSP-1 were shown to inhibit proliferation and to induce apoptosis of murine epithelial ovarian cancer cells (EOC; Russell et al., 2015). Therefore, TSP-1 effects on malignant cells are dependent on receptor expression profiles that are likely to vary between different malignant subpopulations or even depending on their differentiation degrees (Zheng et al., 2015).

Apart from TSP-1-related direct modulation of cancer cell behavior through interactions with membrane receptors, the TSP-1/CD47/SIRPα axis is also strongly implicated in controlling tumor immunity, with both positive and negative roles. A widely held opinion is that tumor cells express high CD47 levels to inhibit phagocytosis by signaling through SIRPα found on macrophages and dendritic cells (DCs; Zhao et al., 2011; Chao et al., 2012; Willingham et al., 2012a). Accordingly, restoration of CD47 expression in CD47-deficient leukemia cells increases xenograft aggressiveness (Jaiswal et al., 2009). To date, CD47/SIRPα is the only known negative regulator of phagocytosis at the immunological synapse and it is known to play an important physiological role in maintaining hematopoietic cells and platelets homeostasis (Olsson et al., 2005; Sick et al., 2012). In addition, CD47:SIRPα interaction may also indirectly promote tumor dissemination through binding of tumor cells to macrophages that reside at the level of potential extravasation sites within the vascular wall (Chao et al., 2011b). Several structural and mutagenesis studies highlighted that according to their respective spatial configuration within CD47 extracellular domain, the TSP-1 and SIRPα interaction sites may not be redundant (Floquet et al., 2008; Hatherley et al., 2008; Jeanne et al., 2015; Soto-Pantoja et al., 2015). However, direct binding assays provided contradictory evidence as both TSP-1 and a function-blocking CD47 antibody inhibit CD47:SIRPα interaction (Isenberg et al., 2009a). Furthermore, recent studies underlined that TSP-1 may also interact with SIRPα (Yao et al., 2014), thus accentuating the impression that the above-mentioned studies asserting an essential contribution of the CD47:SIRPα “don’t eat me” signal remains incomplete, especially as none of this work was done controlling the absence or presence of TSP-1.

Aside from the previously exposed modulation of innate immunity by the CD47/SIRPα antiphagocytic axis, TSP-1 interaction with CD47 existing on immune cells mostly inactivates antitumor adaptive immunosurveillance. Indeed TSP-1 was shown to directly inhibit TCR-mediated T cell activation (Li et al., 2001) by engaging CD47 (Li et al., 2002). Secreted TSP-1 that binds CD47 on T cells inhibits both the NO/cGMP pathway (Ramanathan et al., 2011) and H_2_S signaling (Kaur et al., 2015), therefore concomitantly resulting in an homeostatic inhibitory role of TSP-1:CD47 interaction on T-cell activation. On the other hand, there are cross-talks between the above described mechanisms and VEGF signaling in T cells. Thus, CD47 ligation by TSP-1 inhibits VEGFR2 phosphorylation hence limiting VEGF-induced inhibition of T cell proliferation and TCR signaling (Kaur et al., 2014). Otherwise, TSP-1 binding to CD47 also inhibits differentiation of naïve T cells into Th1 (Bouguermouh et al., 2008), whereas Tregs formation is induced by promoting Foxp3 transcription factor expression (Grimbert et al., 2006; Baumgartner et al., 2008). In cancer, CD47 blockade was shown to enhance antitumor immunity by stimulating CD8+ cytotoxic T cells (Soto-Pantoja et al., 2014). In combination with ionizing radiotherapy that enhances T cell antitumor immunity (Demaria and Formenti, 2012), CD47 blockade in effector T cells is therefore sufficient to inhibit tumor growth, thus offsetting the widely spread opinion that CD47 blockade anticancer effects are attributed to phagocytosis of cancer cells by macrophages. CD47 signaling also regulates natural killer (NK) and DC functions that orchestrate adaptative immunity, leading to tolerogenic signals toward tumor under TSP-1 ligation (Kim et al., 2008; Weng et al., 2014).

Considering these contradictory data, one should realize that TSP-1 roles in cancer progression and metastatic dissemination are complicated and intricate, often leading to paradoxical signals. Indeed, for the purpose of designing new therapeutics, one should bear in mind that several ECM soluble factors and/or cell-surface receptors could bind simultaneously and act as competitors, or even allosterically influence each other’s binding and signaling. Therefore, it seems obvious that TSP-1 and/or its receptors massive blockade or silencing may lead to inevitable adverse effects, closely related to the pleiotropic nature of matricellular proteins and their ligands. Despite these considerations, a few strategies have shown promising results in animal cancer models and some of them already moved to clinical trials.

## Therapeutic Targeting of TSP-1-Related Signaling

Therapeutic strategies targeting TSP-1 signaling and its CD47/CD36 membrane receptors have already been extensively reviewed over the last 5 years (Belotti et al., 2011; Henkin and Volpert, 2011; Sick et al., 2012; Soto-Pantoja et al., 2013b). Thereby, this review is not meant as a comprehensive overview, but rather as a snapshot of current pre-clinical to clinical developments. The range of new therapeutic methods support the sharply expanding interest in targeting TSP-1-related signaling with a view to regulating its function during cancer progression. After describing the most advanced strategies (summarized in **Table 1**) as well as their benefits and limitations, we will discuss more original and sophisticated approaches which aim at modulating TSP-1/CD47/CD36 signalization either directly or indirectly in order to provide an overall anticancer response. Then we will consider future directions and treatment optimizations with the objective of improving further clinical outcomes.

**Table 1:** Therapeutic strategies targeting TSP-1-related signaling.

Compound	Origin/sequence	Target	Stage	Reference
**CD47-blocking mAb**		CD47	Phase 1	clinicaltrials.gov (four trial studies ongoing)
**CD47 antisense morpholino**		CD47	Pre-clinic	Maxhimer et al., 2009
**Peptides**				
4N1/4N1-K	TSP-1 (K-RFYVVMWK-K)	CD47	Pre-clinic	Kalas et al., 2013
7N3	TSP-1 (FIRVVMYEGKK)	CD47	*In vitro*	Maxhimer et al., 2009
PKHB1	i.e., 4N1K, with D counterparts for N- and C-terminal lysines	CD47	Pre-clinic	Martinez-Torres et al., 2015
TAX2	CD47 (CEVSQLLKGDAC)	TSP-1	Pre-clinic	Jeanne et al., 2015
Psap-derived peptide	Prosaposin (DWLPK)	TSP-1 upregulation in Gr1^+^ cells	Pre-clinic	Catena et al., 2013
**TSP-1 recombinant fragment**	**TSP-1**			
3TSR	Type 1 repeats	CD36	Pre-clinic	Zhang et al., 2005
3TSR/TRAIL fusion protein	Type 1 repeats, TRAIL	CD36 and TRAIL receptor	Pre-clinic	Choi et al., 2015
**Peptidomimetics**				
ABT-526 (Abbott)	TSP-1 type 1 repeats (GVITRIR)	CD36	Pre-clinic	Rusk et al., 2006
ABT-510 (Abbott)	TSP-1 type 1 repeats (GVITRIR)	CD36	Phase 2	Baker et al., 2008
ABT-898 (Abbott)	TSP-1 type 1 repeats (GVITRIR)	CD36	Pre-clinic	Campbell et al., 2011
CVX-022 (Pfizer)	TSP-1 mimetic + scaffold Ab	CD36	Pre-clinic	Coronella et al., 2009
CVX-045 (Pfizer)	TSP-1 mimetic + scaffold Ab	CD36	Phase 1	Molckovsky and Siu, 2008
**Non-peptide small molecule**				
sm27	TSP-1 type 3 repeats	FGF-2	Pre-clinic	Taraboletti et al., 2010
**Others**				
Trabectedin (ET-743, Yondelis)	Marine natural product	TSP-1 upregulation by tumor cells	Approved	Monk et al., 2012; Dossi et al., 2015
Velcro-CD47 (N3612)	CD47 extracellular domain	SIRPα	*In vitro*	Ho et al., 2015

### Antibody Blockade and Gene Therapeutics

The use of monoclonal antibodies (mAbs) is an obvious way to therapeutical target cell-surface receptors. Considerable efforts have focused on developing CD47-targeting mAbs to block the CD47/SIRPα antiphagocytic pathway established between tumor cells and immune cells. Such CD47-blocking mAbs were shown to be effective by allowing the decrease of tumor burden in several preclinical cancer models including acute myeloid leukemia (Majeti et al., 2009), lymphoma (Chao et al., 2010) and osteosarcoma (Xu et al., 2015). While the decrease in tumor growth is mainly attributed to enhanced tumor cell clearance by macrophages under CD47:SIRPα disruption (Willingham et al., 2012a), other studies have noted that alternative mechanisms may explain the antitumor activities of CD47-blocking antibodies. Particularly, the use of intact IgG (such as B6H12 anti-CD47 Ab) in the previously mentioned *in vivo* experiments may also induce Fc-mediated cytotoxicity (Zhao et al., 2011). Of note, one of the CD47-blocking antibody that reduced tumor growth (clone miap410; Willingham et al., 2012b) raised doubts as to its ability to block CD47:SIRPα interaction (Han et al., 2000; Willingham et al., 2012b). Altogether, these data suggest that increased macrophage phagocytosis is not sufficient to explain antitumor activities of CD47-targeting mAbs and that other actors are involved (Soto-Pantoja et al., 2012a; Zhao et al., 2012). Particularly, *in vitro* and *in vivo* studies have shown that macrophages are able to prime an effective CD8+ T cell response following anti-CD47 treatment-mediated phagocytosis of cancer cells, by concomitantly inducing a reduction in regulatory T cell population (Tseng et al., 2013). To date, at least four first-in-man phase 1 clinical trials considering anti-CD47 humanized mAbs are underway, according to clinicaltrials.gov website (identifiers NCT02216409, NCT02447354, NCT02488811, and NCT02367196). Given the ubiquitous expression of CD47, systemically administered anti-CD47 mAbs will inevitably come across a huge number of CD47 copies on red blood cells (RBCs). To avoid phagocytic-induced excessive reduction in erythrocytes count, it has been suggested to use a priming-dose of anti-CD47 that would result in “aged” RBCs removal and subsequent erythropoiesis stimulation (McCracken et al., 2015). Such suggestion is obviously questionable, as many other clearance mechanisms are known to be preponderant in triggering removal of senescent RBCs (Lutz and Bogdanova, 2013). One should note that experiments considering CD47 targeting agents in mice did not induce any significant anemia, which also runs counter to a major role for antiphagocytic “don’t eat me” signal disruption in these studies. Besides, CD47 plays fundamental physiological roles by limiting NO signaling in RBCs, platelets, and endothelium (Soto-Pantoja et al., 2015). Indeed, CD47 antibody targeting may affect NO pathway modulation and subsequent angiogenesis regulation, since a commonly used CD47-blocking antibody (clone B6H12) was previously shown to concomitantly disrupt both TSP-1:CD47 and CD47:SIRPα interactions (Isenberg et al., 2009a). As pre-clinical data suggests that high circulating TSP-1 levels produced by tumor stroma may indirectly increase tumor perfusion while decreasing peritumoral and systemic blood flow, CD47-targeting mAbs are therefore likely to counteract these effects through regional stimulation of NO signaling (Isenberg et al., 2008b, 2009b). On the other hand, anti-CD47 antibodies may also interfere with CD36-mediated modulation of NO signaling, as CD47 is required for CD36 activation under TSP-1 ligation (Isenberg et al., 2006). According to this, systemic administrations of anti-CD47 mAbs for cancer treatment would probably lead to severe adverse events such as hypertension and thrombosis. Therefore, we are not fully persuaded by the use of CD47 antibodies as an alternative to current anticancer drugs, while their local use is much more promising for instance in ischemia prevention (Lin et al., 2014).

As RBCs have prolonged circulating lifetimes without any membrane protein turnover (Mohandas and Gallagher, 2008), other groups have suggested that acute genetic modulation of CD47 expression may represent a surrogate to some of the antibody-based strategies side-effects. Indeed CD47 antisense morpholino potently reduced tumor burden in patient-derived hepatocellular carcinoma xenografts (Lee et al., 2014). This study highlighted that the use of morpholino against CD47 mRNA may be of particular interest in combination with conventional chemotherapy as it potentialized the effects of doxorubicin. In the context of syngeneic melanoma allografts, morpholino suppression of CD47 expression induced only a modest decrease of tumor growth (Maxhimer et al., 2009). There again, more beneficial effects were reached when combining morpholino treatment with radiotherapy in the same allograft model. It should be noted that similar inhibition of tumor growth is observed when irradiating TSP-1 null mice, thus suggesting that anticancer targeting of TSP-1:CD47 interaction would be of a greater relevance than disrupting CD47:SIRPα (Isenberg et al., 2008c; Soto-Pantoja et al., 2013b). Accordingly, TSP-1 silencing in DCs by shRNA interference exhibited antitumor effects in a bladder cancer syngeneic model, by increasing tumor-infiltrating CD4+ and CD8+ T cells (Weng et al., 2014). There again, TSP-1 wide-spread silencing may be a double-edged sword for cancer therapy as TSP-1 exerts opposite effects in endothelial cells and DCs. Therefore, the use of TSP-1 recombinant fragments or small antagonistic molecules may be of a better interest.

### TSP-1-derived Peptides, Recombinant Fragments, and Mimetics

Several synthetic peptides derived from the C-terminal domain of TSP-1 were early identified as containing a critical VVM motif proposed to be essential for CD47 binding (Gao and Frazier, 1994). Among them, the widely used 4N1 (^1016^RFYVVMWK^1024^) and 7N3 (^1102^FIRVVMYEGKK^1112^) are able to reproduce some of TSP-1-mediated biological effects in *in vitro* models (Rath et al., 2006a; Maxhimer et al., 2009). However, 4N1 should no longer be considered as a CD47-specific targeting agent as several studies pointed out 15 years ago its ability to induce cellular responses in a CD47-independent fashion (Tulasne et al., 2001; Barazi et al., 2002). In addition, there is a dearth of convincing *in vivo* data concerning these peptides, probably due to moderate affinity to CD47 and poor pharmacokinetic properties, thus requiring high dose treatments. Indeed repeated administrations of an extended version of the 4N1 peptide named 4N1K (K-^1016^RFYVVMWK^1024^-K) induce only modest changes in tumor growth (Kalas et al., 2013), while 4N1K exhibits low stability in plasma. Consideration of 4N1K as a CD47 agonist is all the more controversial as its VVM motif is actually buried within a hydrophobic β-strand arrangement of C-terminal TSP-1, therefore, avoiding accessibility to CD47 without significant conformational changes (Kvansakul et al., 2004). However, normal mode analysis and energy minimizations helped to identify large amplitude motions of TSP-1 signature domain, leading to opening of the hydrophobic cleft and allowing solvent exposure of the 4N1 sequence (Floquet et al., 2008). Some studies highlighted differences in 4N1K-induced responses between CD47+/+ and CD47-/- cells or considering CD47 blocking mAbs (Fujimoto et al., 2003; McDonald et al., 2004), which remains unexplained in view of the above-mentioned studies demonstrating 4N1 non-specificity. Despite these considerations, others have suggested that some of the *in vitro* effects of 4N1K are likely to be due to its hyper-adhesive nature rather than its interaction with CD47, particularly as CD47-deficient cells are able to bind immobilized 4N1K (Leclair and Lim, 2014). Interestingly, a recently identified serum-stable analog of 4N1K named PKHB1, in which natural L-amino acids are replaced by their D counterparts, was demonstrated to induce a twofold reduction in human chronic lymphocytic leukemia xenografts growth (Martinez-Torres et al., 2015). Nevertheless, caution should be observed about such TSP-1-derived CD47 agonists as they might also induce adverse inhibitory effects on host DCs immunity (Weng et al., 2014).

Among TSP-1 multiple domains, the main antiangiogenic sequences are thought to reside within the type 1 repeats involved in CD36 binding (Belotti et al., 2011). Indeed recombinant 3TSR fragments potently inhibit tumor growth in both syngeneic melanoma and orthotopic human pancreatic carcinoma models (Miao et al., 2001; Zhang et al., 2005). Subsequently, TSP-1-derived peptidomimetics were developed and even reached phase 2 clinical trials. ABT-526 (Abbott Laboratories) was the first to be described as a modified peptide based on the GVITRIR sequence of the second TSP-1 type 1 repeats (Haviv et al., 2005), and yield impressive disease regression without any significant adverse effects in tumor-bearing dogs (Rusk et al., 2006). Thereafter a more soluble enantiomer with better PK/PD profile, named ABT-510, entered clinical trials. After showing relevant PK properties in phase 1 trials (Hoekstra et al., 2005; Gietema et al., 2006), ABT-510 failed to give clear evidence of efficacy in phase 2 and led to severe adverse events such as thrombosis and pulmonary embolism (Ebbinghaus et al., 2007; Baker et al., 2008). ABT-510 is consequently no longer tested in clinical development, however, a second-generation mimetic named ABT-898 has recently emerged with improved therapeutic activity in dogs with soft tissue sarcoma (Sahora et al., 2012). While ABT-898 treatment efficiently allowed the regression of established ovarian tumors in mice (Campbell et al., 2011), it has not entered human development so far. Conjointly, CVX-22 and CVX-045 (Pfizer) were developed fusing TSP-1-derived peptidomimetics with a proprietary scaffold antibody (Levin et al., 2007; Coronella et al., 2009). While CVX-045 showed efficacy in tumor xenografts by reducing MVD and increasing necrotic cores (Li et al., 2011), only limited benefits were observed during phase 1 clinical trials in association with severe adverse events (Molckovsky and Siu, 2008), which probably explains why such “peptibodies” have been discontinued from Pfizer pipeline in 2014 (Rader, 2014).

### Original Strategies and Current Developments

Aside from peptidomimetics based on sequences from the type 1 repeats, sm27 is a non-peptide small molecule mimicking the FGF-2 binding site located in the type 3 repeats of TSP-1 (Taraboletti et al., 2010) that exhibits *in vitro* and *ex vivo* antiangiogenic properties (Colombo et al., 2010). Since 2010, several computational studies have been conducted aiming to optimize sm27:FGF-2 binding dynamics (Pagano et al., 2012; Meli et al., 2014), and newly designed derivatives will presumably be evaluated *in vivo* in future experiments. More recently, we characterized a cyclic peptide derived from CD47, named TAX2, that directly binds TSP-1 to antagonize TSP-1:CD47 interaction (Jeanne et al., 2015). TAX2 administration led to a decrease in viable tumor volume in melanoma allograft and potently inhibited pancreatic carcinoma xenograft growth, together with a disruption of tumor-associated vascular network. *In vitro* studies using CD36 blocking mAbs indicated that the unpredicted antiangiogenic properties of TAX2 are likely to be mediated by CD36 activation. According to the TAX2 proposed mechanism of action, such peptide may induce a TSP-1 binding switch from CD47 to CD36. Appropriately, TAX2 antitumor effects are consistent with those observed using TSP-1 recombinant fragments targeting CD36 in similar experimental models (Miao et al., 2001; Zhang et al., 2005). The use of recombinant 3TSR as a CD36-activating treatment recently showed promising results in preclinical models of glioblastoma and ovarian carcinoma (Choi et al., 2015; Russell et al., 2015), therefore it seems relevant to assess the therapeutic potential of TAX2 in the context of such pathologies. In addition, ABT-898 was shown to be especially potent in the female reproductive tract (Campbell et al., 2011), and CD47 was early considered as an ovarian tumor marker (Campbell et al., 1992). Through its original mechanism of action which supposes concomitant disruption of TSP-1:CD47 interaction and enhancement of CD36 activation by TSP-1, TAX2 may inhibit tumor progression while limiting many of the undesired side effects of broadly inhibiting important physiological functions of CD47. Indeed, as TAX2 was designed to target TSP-1 specifically at the CD47 binding site, both TSP-1 and CD47 are presumed to remain free to interact with their respective alternative ligands. Nevertheless, some putative side-effects of using TAX2 as an anti-cancer agent still need to be explored, particularly as TSP-1 interaction with CD47 and/or CD36 is also known to modulate platelet aggregation (Isenberg et al., 2008d). While ABT-510 lack of efficacy in clinical trials is likely due to its inability to mimic the activity of full-length TSP-1 (Ebbinghaus et al., 2007; Markovic et al., 2007), we are convinced that original strategies, viz. the use of TAX2 or the identification of new inhibitors that would target pathway leading to TSP-1 repression, may provide realistic treatment alternatives by finely controlling full-length protein signaling. Interestingly, TAX2 was shown to inhibit endothelial cell cGMP production under NO stimulation. According to our assumptions, TAX2 may target the NO/cGMP pathway downstream from eNOS through stimulation of TSP-1:CD36 interaction. Hence, unlike bevacizumab or other VEGF-targeting blockbuster drugs, TAX2 may also inhibit downstream signals resulting from angiogenic signals other than VEGF such as NO production by stromal cells (Roberts et al., 2007). Currently, further work is being done to improve TAX2 translational potential.

Other molecules were also shown to cause an overall anticancer response by involving the action of TSP-1. Indeed trabectedin (ET-743, Yondelis), a marine natural product approved as a second-line treatment of recurrent ovarian cancer (Monk et al., 2012), exhibits antiangiogenic activities by upregulating tumor cell expression of TSP-1 (Dossi et al., 2015). Besides, a five-amino acid peptide derived from prosaposin (DWLPK) was recently shown to inhibit lung metastatic colonization through upregulation of TSP-1 in Gr1^+^ myeloid cells (Catena et al., 2013). Therefore, direct stimulation of TSP-1 or even strategies that indirectly increase bioavailable TSP-1 within the pulmonary microenvironment could therefore represent a relevant translational antimetastatic approach. However, attenuation of NO and activated CD47 corroborate with pulmonary hypertension (Xu et al., 2004; Bauer et al., 2012) while TSP-1 is a characteristic component of coronary atherosclerotic plaques (Riessen et al., 1998). Therefore, inducing TSP-1 may lead to cardiovascular complications, especially in the lung (Rogers et al., 2014). In addition, caution should be exercised in generalizing the benefits in other host organs, as they might be dependent on the cytokine environment. Particularly, Lee and collaborators reported that ADAMTS1-mediated processing of TSP1 into antiangiogenic fragments occurs differently for liver and lung metastases (Lee et al., 2010).

Besides the above-described TSP-1-related therapeutic strategies, much has also been done to propose alternative methods to target CD47/SIRPα signaling. While current approaches have principally targeted the ubiquitously expressed CD47, thus inevitably leading to off-target effects, a novel engineering development has recently emerged aiming to target SIRPα specifically. The so-called “Velcro-CD47” (N3612) consists of a high affinity variant of the human CD47 extracellular domain extended at the N-terminus with a short three amino-acid peptide in order to increase binding affinity to SIRPα (Ho et al., 2015). Velcro-CD47 already proved its ability to enhance macrophage phagocytosis of tumor cells *in vitro* and to target the monocyte subpopulation specifically, and its putative anticancer efficacy will be further evaluated in pre-clinical models.

### Future Directions

In order to reach an optimal control of tumor progression, future directions will aim to associate innovative approaches targeting TSP-1/CD47 and TSP-1/CD36 signaling with existing anticancer treatments. Indeed morpholino suppression of CD47 expression was shown to markedly increase radiation-induced delay in tumor growth considering two syngeneic models of melanoma and squamous cell carcinoma (Maxhimer et al., 2009; Ridnour et al., 2015). While it sensitizes the tumor to ionizing radiation, CD47 deficiency concomitantly confers radioprotection to normal tissues through activation of autophagy (Soto-Pantoja et al., 2012b). This may be of particular interest in the field of blood cancer treatment with the aim of minimizing the adverse effects of total body irradiation, especially as morpholino-induced CD47 gene silencing was demonstrated to preserve circulating peripheral blood cells and to protect gastrointestinal tissue from ionizing radiation (Soto-Pantoja et al., 2013a). Therefore, future studies will determine the appropriate strategies targeting CD47 with the purpose of radiomitigation, with the potential of being translated into clinical practice.

CD36-activating 3TSR treatment efficacy was also evaluated on top of conventional chemotherapy. While intermittent bursts of MTD chemotherapy are currently considered in the treatment of ovarian cancer, combination with 3TSR may facilitate the uptake of drugs delivered at low-dose MET scheduling in order to reach higher tumor regression rates in patients with advanced EOC (Russell et al., 2015). Indeed combination of 3TSR with carboplatin and paclitaxel MET chemotherapy considerably promotes survival in a syngeneic murine model of EOC. Interestingly, 3TSR is more effective than ABT-598 in this model, thus supporting the concept that the full function of the type 1 repeats cannot be mimicked by a single short peptide (Campbell et al., 2011). Another promising strategy consists of combining the antiangiogenic property of 3TSR with the pro-apoptotic TRAIL in order to target both tumor and tumor-associated vessels (Ren et al., 2009). Such 3TSR/TRAIL fusion protein was recently demonstrated to improve survival of mice bearing intracranial human glioblastoma xenografts, therefore, suggesting a potent translational potential of 3TSR/TRAIL therapies into clinics (Choi et al., 2015).

Finally, TSP-1 peptidomimetics may be considered not only for their direct therapeutic use, but also to enhance the therapeutic delivery of cytotoxic drugs. Notably, a D-reverse peptide derived from the native KRFKQDGGWSHWSPWSSC motif within the TSR of TSP-1 was first demonstrated to inhibit breast tumor growth in a mouse xenograft model (Guo et al., 1997). More recently, an aspartimide analog based on the same TSP-1 sequence was shown to potentiate the activity of doxorubicin in colon carcinoma xenografts. Indeed, such a peptide is able to support the adhesion of doxorubicin-containing liposomes to both tumor cells and endothelial cells, thus leading to increased antiproliferative and antiangiogenic activities (Rivera-Fillat et al., 2010).

## Conclusion

To date, mAbs targeting CD47 are the best advance toward clinical development and much interest is accorded to massive anti-CD47 blocking strategies, even within non-scientific skilled communities. Accordingly, a growing number of almost sensational reports excessively praise the therapeutic potential of CD47-targeting anticancer immunotherapies on social networks, video sharing platforms or popular-science writings (Williams, 2012; Foley, 2013), probably with a promotional and fund raising purpose. Noteworthy work of Weissman and collaborators in immunodeficient mice has proved preclinical efficacy of anti-CD47 mAbs in a wide range of xenograft models including leukemia (Chao et al., 2011a), lymphoma (Chao et al., 2011b), multiple myeloma (Kim et al., 2012), and several solid tumors (Edris et al., 2012; Willingham et al., 2012a). However, we are deeply convinced that genetic ablation or antibody blockade of CD47 may not represent a fully satisfying anticancer therapeutic alternative due to adverse effects and/or concomitant attenuation of beneficial functions, and that a more nuanced picture could be exposed to cancer patients. Indeed, massive extinction of any protein/receptor/signaling pathway might lead to adverse effects and resistance, while more accurate strategies are needed to regain the baseline. In particular, a global vision of the numerous molecular and cellular actors involved should be adopted when considering matricellular proteins and their receptors in anticancer drug development. Anti-CD47 mAbs could offer clear benefits in the treatment of cardiovascular diseases, however their use as anticancer drugs is likely to encounter the same limitations as bevacizumab, i.e., hypertension, thromboembolism, and tumor recurrence (Gil-Gil et al., 2013). While genetic modulation of CD47 expression could represent an alternative to antibody-based strategies, further clinical development of previously described morpholino-based approaches may require repeated administrations of high doses, due to morpholino oligonucleotides poor cell and tissue uptake as well as their rapid renal clearance (Moulton and Moulton, 2010). Besides, siRNA and miRNA-based strategies may provide viable alternative to morpholino-based CD47 silencing (Wang et al., 2015).

In our opinion, future research should focus on small molecules that allow a finer and more accurate regulation, thus leading to adequate responses and limited adverse effects. Among the variety of innovative approaches, peptides represent a fast-growing class of new therapeutics (Diao and Meibohm, 2013) and many structural modification strategies have been developed recently to improve their performance as drugs (Di, 2015). The combination of such cutting-edge strategies with conventional anticancer agents will help optimize dosing schedules, whose influence on resistance acquisition is often under evaluated, particularly among anti-VEGF approaches (Clarke and Hurwitz, 2013). Looking ahead, original and selective TSP-1-related antireceptor strategies could improve long-term benefits by overcoming many undesired effects. The next challenges will concern the translation of these small molecules into the clinic, as well as the identification of optimal combinatorial strategies with standard chemotherapy and radiotherapy.

## Author Contribution

CS and LM contributed to write the manuscript; AJ and SD wrote the manuscript; SD supervised the work.

## Conflict of Interest Statement

The authors declare that the research was conducted in the absence of any commercial or financial relationships that could be construed as a potential conflict of interest.

## Abbreviations

3TSR: three thrombospondin-1 type 1 repeats
ECM: extracellular matrix
EOC: epithelial ovarian cancer
FGF-2: fibroblast growth factor-2
MET: metronomic
MTD: maximum tolerated dose
PD: pharmacodynamic
PK: pharmacokinetic
RBCs: red blood cells
SIRPα: signal regulatory protein alpha
TCR: T cell receptor
TRAIL: tumor necrosis factor-related apoptosis-inducing ligand
TSP-1: thrombospondin-1
VEGFR2: vascular endothelial growth factor receptor 2
